# Language games and blurry terminology: Can clarity enhance athlete development?

**DOI:** 10.3389/fspor.2023.1150047

**Published:** 2023-04-17

**Authors:** Kathryn Johnston, Alexander B. T. McAuley, Adam L. Kelly, Joseph Baker

**Affiliations:** ^1^ Kinesiology and Health Science, York University, Toronto, ON, Canada; ^2^ Research Centre for Life and Sport Sciences (CLaSS), School of Health Sciences, Birmingham City University, Birmingham, WMD, United Kingdom

**Keywords:** athlete development, measurement precision, athlete selection, talent identification, careful language use

## Abstract

This perspective focuses on the need for researchers and practitioners to carefully consider the clarity and consistency of their language in the context of athlete development. Evidence supporting a lack of congruency in the way certain terms and expressions are defined, understood, and operationalized continues to accumulate, highlighting the importance of this area for sport stakeholders and the potential looming crises. In systems that regularly rely on precision and accuracy, it will be critical that all involved in the co-creation of knowledge generation and application carefully consider terms that may further complicate athlete development practices. We highlight some potentially blurry terms and draw attention to potential avenues for future research.

## Introduction

“…*words are the tools with which we work… Everything depends on our understanding of them*” **—**US Supreme Court Justice, Felix Frankfurter

In 1958, German philosopher Ludwig Wittgenstein suggested that linguistic confusion (i.e., the misuse and misunderstanding of language) was the root of all philosophical problems. He (1) further noted that “language games” (i.e., the use of language and the actions into which it is woven) are an inevitable facet of human behaviour. Individual perceptions and interpretations of various terminology^1^ also exist as language is so heavily intertwined with one's experiences, which are profoundly shaped by sociocultural backgrounds, geographic locations, and education, among other variables. This inconsistency can have wide-ranging implications on research conceptualisation, sample descriptions, measurement precision, and practical applications due to differences in how terminology is operationalized (2).

The field of athlete development, which pertains to the identification, development, and selection processes of sporting populations, is not immune to these language challenges. Various undefined or vaguely defined terminologies have been recognized for causing confusion and contradiction in both research and practice. These “blurry” (i.e., unclear or poorly defined) words are believed to be one of the main limitations of research and knowledge translation (3). At a time of sport datafication, characterized by the increased recording, analyzing, and interpreting of sport information (4), this is perhaps surprising. While this datacentric view of sport can offer many potential benefits (e.g., the possibility of hyper-specific training and development plans for athletes (5, 6), enhanced engagement for fans (7), and improved tactical decisions for coaches (8, 9), a deep concern remains; namely that the critical language used by coaches, researchers, and other sport stakeholders is often vague, nebulous, and lacking appropriate nuance. In our own areas of research (spanning talent identification, athlete selection, athlete development), some of these blurry terms have recently been exposed, such as “talent” (10), “elite” (11), “coach's eye” (12), “sampling” (13), “early specialization” (14), “mental skills” (15) and “positive youth development” (16). This work suggests the field is becoming increasingly aware of how problematic these words are and illuminates how difficult it can be to refine, replace, and/or remove such terms. This difficulty may be related to the frequent (mis)use of the terms found in discourse (e.g., policy or guiding documents), and further shared colloquially (e.g., in certain communities and groups) and discussed through media outlets.

## Striking a balance between vagueness and conceptual clarity

“*Among other things, the message is that we need to command a clear view of the use of our concepts, [and] be aware of the danger (and potential) of words…*” (3) (*p*. 90)

In some disciplines of sport science, such as sport psychology and sport sociology, it may be unrealistic to expect the same level of specificity and exactness we see in other domains. This is most likely a result of how “open” a system becomes (i.e., chaotic and dynamic) when considering a person's “performance” which encapsulates the interaction of social, genetic, environmental, cultural, spiritual, and psychological components, compared to disciplines like physiology and biomechanics, which are considered more “closed” systems (i.e., often objectively informed). It is also likely that some degree of blurriness could be seen as advantageous for athlete selection and development reasons. Take for example a coach whose career hinges on the selection of a successful cohort of athletes. With more vague and blurry selection criteria, this may create more “degrees of freedom” for him/her/them to defend the selection decisions they make. This notion also speaks to the art and science of coaching—where both scientific rigor coupled with creative and dynamic liberties are believed to be critical for coaching excellence (17, 18). However, there is an increasing push to clearly define and defend selection criteria, at least in high performance settings (19).

With this in mind, it might be advantageous to strike a balance between accepting conceptual vagueness and delineating sharp boundaries to quantify certain terms or phenomena. Determining *which* terms require greater focus, delineation, and precision is difficult; however, it could be argued that any term involving measurement requires precision. For example, “talent” is a blurry term, and incredibly hard to define (see (10, 20, 21)), yet it is often at the root of decision-making in many sport systems (e.g., when a team conducts a formal “talent identification camp”). Researchers and practitioners would undoubtedly benefit from increased conceptual clarity in the hope of making more accurate talent measurements and selections.

Perhaps it would be beneficial to even consider some terms on a scale or continuum instead of being binary and absolute (22). It is human nature to think of concepts and constructs in this rigid way (23), but sport rarely operates in such a fashion. In this sense, there could be value for terms that cannot be defined in a simple dichotomy to be considered relative to a scale or spectrum (e.g., early specialization; see (14) for a review). This type of theoretical and philosophical trade-off has been proposed in other fields, such as ecology, ecological economics, and computing (see (24–29) for examples), and while it is beyond the scope of this article to adequately present, let alone resolve, the disputes between philosophy and cognitive science, an emphasis should be placed on moving away from such blurry terms in the pursuit of making higher-quality decisions for athlete selection.

**Figure 1:**
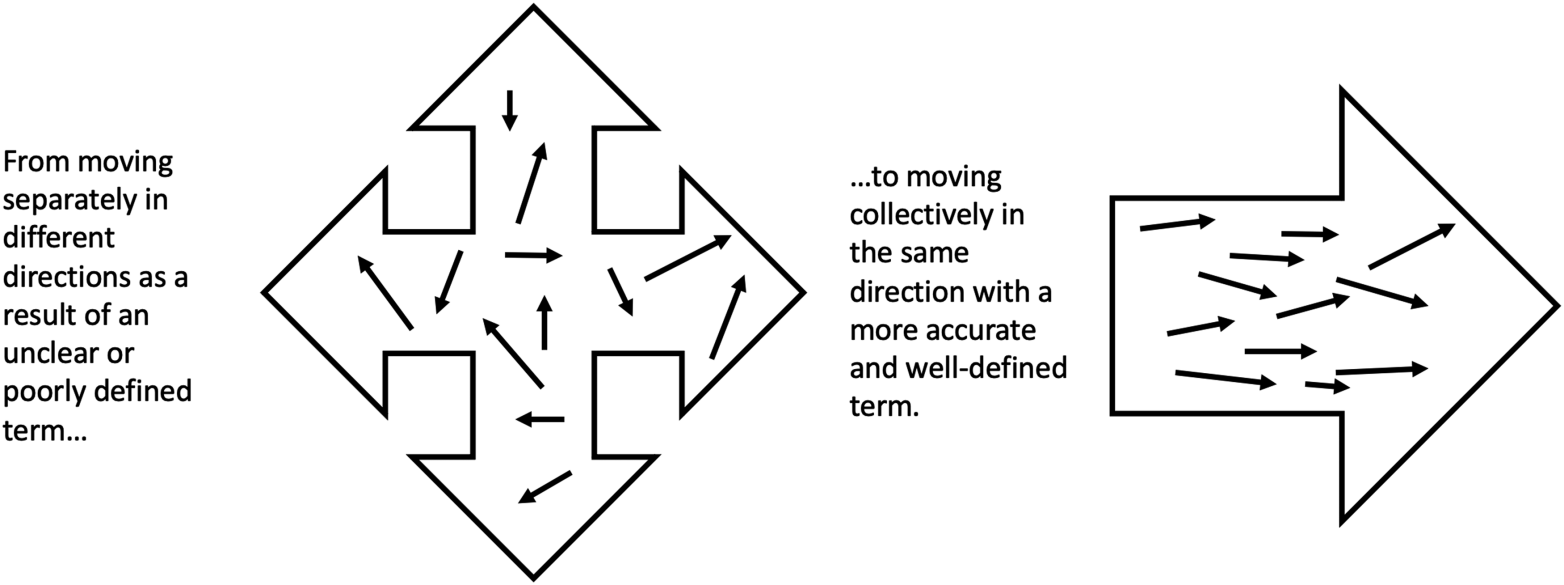
A visual depiction of how individual perceptions of terms may result in movement in various directions, but alignment can lead to a unified movement forward (adapted from (31)).

It is also important to acknowledge that organizational alignment, may be more critical than universal agreement. For example, player scouts (i.e., sport staff who assess athletes for their suitability for a given team) should have alignment in how they define certain aspects of sport (30). If two scouts hold substantially different definitions of “grit”, then depending on which scout performs an assessment on an athlete, it will impact the way the scout reports and shares that information with others, thus impacting the way others perceive that athlete’s grit. The organization could thus benefit from creating criteria or scales for what “grit” is and how it is portrayed by athletes, so there is greater organizational alignment. In other words, concepts do not always need to be rigidly defined, but the goal should be to improve the precision of measurement as much as possible over time as concepts become clearer. This will help stakeholders and organisations move collectively in the same direction (see Figure 1). For example, a recent scoping review on the conceptualization and measurement of positive youth development in sport revealed 243 unique operational definitions (16). This is clearly problematic, and since researchers and practitioners have different views of what the term and definition mean and how it is operationalised in research and practice, creating alignment is necessary to avoid such confusion and contradiction.

## Improving conceptual clarity?

The reality is, language affects all stakeholders—the person who uses the language, the sport system he/she/they operates within, the athletes hearing the language, along with parents, other colleagues, and the list goes on. What we have learned from work conducted in the fields of psychology, language, and education (see (32) for an example), is that language can shape perceptions (i.e., belief about one's abilities and capabilities), goals (i.e., the type and nature of what is being strived for), and actions (i.e., the accompanied behaviours). For instance, beliefs about talent have the potential to promote feelings of learned helplessness in contexts where athletes believe a lack of talent is insurmountable for their long-term aspirations (i.e., Pygmalion effects) or complacency in contexts where athletes believe their talent assures them future success and subsequently decreases their effort and motivation to improve (i.e., Crown Prince Effects (33). For this reason, it is important to carefully examine terms that could be considered blurry and, therefore, less useful than they might be appear.

Fortunately, determining a term's blurriness can be done through a process of identifying its definition within the contexts/situations that it is used. Take, for example, administrative staff in a sport organization trying to assess the quality of their talent selection criteria. The staff could attempt to define what “talent” means in the context of the sport, age, and level of athlete as well as within the environmental constraints of the sport system. If consensus, or even alignment cannot be reached on a definition (which seems likely given recent research (17)), this warrants a discussion on the value of “talent” in the system. This alignment is particularly important in an organizational setting, as it has been proposed that organizational alignment (in terms of values, priorities, and goals) may not just be important, but critical for success (34–36). In short, if a term cannot be defined, then how can it be measured, if it cannot be measured, how can it be monitored and improved, if it cannot be monitored and improved, what is its value? Below, we draw attention to three common terms used in athlete development research and practice as examples to help highlight the importance of clarity for stakeholders.

*Elite.* Telling athletes they are “elite” could have important effects on feelings of competence and motivation (37). In this sense, athletes may believe they have reached a certain status that may affect their thoughts about the value of training, how they compare themselves to others and other groups (i.e., on the competition hierarchy), and their ability to access important developmental resources. For instance, during the COVID-19 pandemic, lockdown restrictions were put in place in many countries with titles and labels used to restrict access to key developmental resources. However, due to the Olympic Games in Tokyo, competing athletes considered “elite” were allowed to continue training, without the limitations imposed on “non-elite” athletes. Determining what “elite” meant in this context caused considerable confusion and upset for many athletes. In the United Kingdom, “elite” was seen as broadly applying only to athletes 16 years of age and older, although this varied by sport (Category 1 and 2 youth soccer academies were allowed to continue across all ages) and by gender (no female academies were allowed to continue). It is perhaps unsurprising, that decisions at the policy level around what quantifies “elite” athletes have ripple effects into other aspects of sport. For example, labelling athletes as “elite” in sports science research considers them in a rather homogenous way. This can be misleading for a reader as important accompanying and qualifying information like age, playing level, sociocultural context, may not be included. This leads to further misinterpretations during research syntheses, where athletes may be grouped by their status (i.e., as “elite”) despite being in vastly different contexts.

*Character.* The term “character” has been used as a selection criterion in sport for many years (38). In this context, coaches and selectors look to match an athlete's character traits to the priorities and values of the sport program/system. The term can become particularly blurry when examining the behaviours and mannerisms valued across different countries and sociocultural backgrounds. As an example, cultures that are considered to be individualistic (i.e., where the need of the individual supersedes the need of the group (39)) see behaviours such as asking questions, making eye contact, and challenging concepts to develop understanding as positive. In comparison, within collectivist cultures (i.e., where the needs of the group supersede the need of the individual (39), these same behaviours could be viewed as disrespectful (40). Based on these contexts, and depending on the cultural norms of the coach or selector (i.e., individualistic vs. collectivist), different aspects of a player's “character” could be assessed as either a positive or negative attribute. In a time when sport is working to reduce individual and systemic biases and inequalities (41–44), conceptual clarity may help achieve more equitable organisational structures by being clearer on what terms such as “character” entails.

*Training load*. The term “training load” term is generally used in reference to the physiological demand placed on an athlete, typically measured in terms of time (e.g., number of practice hours), energy expenditure (number of kilocalories burned), and/or stress (e.g., heart rate) (see (45) for more information). While these variables are relatively straightforward to quantify, they are incomplete proxies for overall “training load”. Rarely considered are the “external” (i.e., demands placed on the athlete beyond training/practice and competition), “internal” (i.e., self-imposed demands) loads, and social and psychological loads from within and beyond the training environment that are a direct result of sport participation and training. For example, collegiate level athletes have demands from both their sport and academic careers (46). Arguably, overall training load should consider their academic demands as they are inextricably paired (i.e., without being an academically-eligible student, collegiate-level athletes cannot compete). As Farrow and Robertson (47) noted, using a periodized approach (i.e., systematic variations) to physical training is important, but it must consider additional dimensions of the training demand (both internally and externally), which can be difficult to capture given the complex interaction of an athlete's biological response to training stimuli as well as his/her/their developmental status. It is thus important to consider the context and the value of using a term like “load” more broadly, as it might be perceived as being reductionist and perhaps even disconnected from other critical psycho-social components of the athlete training experiences.

## Moving forward

Athlete development appears to be at a watershed moment, with considerable research energy being spent on clarifying terms and improving measurement precision. Greater clarity in language will have impacts throughout the sport system, from researchers and policy makers to coaches at the frontlines of sport delivery. To help minimize these linguistic challenges, it could be valuable for the field to have a glossary/dictionary of frequently used words where some form of a consensus has been reached. The glossary/dictionary could benefit stakeholders by offering trustworthy, current, and peer reviewed definitions of terms that have been validated (i.e., tested/applied reliably across time and various diverse settings) and accepted (i.e., through a peer-reviewed system, the term was deemed “acceptable” for use) by multiple stakeholders in the field. As with the case of a language dictionary (e.g., the Oxford English Dictionary), the goal would be to create a “go-to” list of terms so that researchers and practitioners could utilize and reference such terms for consistency and clarity.

That said, approaches to create such a tool would undoubtedly come with challenges and obstacles. For example, decisions on “*who*” could/would contribute to such a tool would come with biases. Moreover, decisions on “*what*” definition(s) are included would likely be biased as well. To help reduce the impact of the “*who*” bias, ultimately, multiple stakeholders (e.g., researchers and practitioners) from different perspectives (i.e., spanning sport disciplines, countries, philosophical positions, cultures, competitive levels etc.) would contribute to create the *most appropriate* definition for that *point in time* based on the *current state of knowledge*. One approach could include using a consensus statement. The most popular of the consensus statement approaches includes the Delphi and “modified Delphi” designs (48), where groups of experts are asked their opinions on a particular issue/phenomenon (49) (see (50) for this approach applied in the context of sport). While these can be powerful tools for informing sporting policy and practice, there are important considerations. As noted by Blazey and colleagues (48), it is important for such consensus statements to carefully consider *who* might be missed in the consensus process, *who* may be coerced to agree on terms, and *how* can such statements capture the rich discussion that is had before the consensus has been reached. The authors also note that in many consensus statements, there is inadequate reporting of methods to allow for knowledge synthesis, drawing into question the rigor in the consensus process. To help combat this, Blazey and colleagues recommend a) reporting the criteria for the selection of “experts”, b) reporting the selected panel's participant details (including their expertise on the topic in question), c) defining “a priori” what level of agreement is allowed and whether discordance will be reported, d) acknowledging opposing opinions, and e) externally validating results.

To help minimize the “*what”* bias, an emphasis should be placed on this being a “living document”– requiring updating as new evidence emerges, and relying on crowd sourcing to capture as many perspectives as possible. In this case, there would be not one, but multiple definitions that consider various philosophical perspectives on the meaning of language within different contexts (e.g., Wittgenstenian (1), Kuhnian (51), Popperian (52)) for researchers and practitioners to consider and reference. An alternative method to the consensus statement may include a text-mining analysis or possibly a survey of researchers and practitioners to identify what topics and terminology are the “blurriest”. This could lead to a citation network analysis, which would be a useful step to establish the most impactful/cited authors and articles on a specific topic or terminology to determine what may be the most influential interpretations.

## Final remarks

In this perspective, we acknowledged frequent use of “blurry” terms may be limiting the quality of evidence due to measurement imprecision. Amongst a growing field of research and practice in athlete development, it will be paramount for the sake of all stakeholders to remain attentive to the language they use. We believe the field is at a critical juncture where striking a balance between vagueness and conceptual clarity will be a necessity to advance the field forward in the right direction.

## Data Availability

The original contributions presented in the study are included in the article/Supplementary Material, further inquiries can be directed to the corresponding author.

## Footnotes

^1^Terminology is used to capture words, phrases, concepts, and constructs.
